# Experiences of hearing voices: analysis of a novel phenomenological survey

**DOI:** 10.1016/S2215-0366(15)00006-1

**Published:** 2015-04

**Authors:** Angela Woods, Nev Jones, Ben Alderson-Day, Felicity Callard, Charles Fernyhough

**Affiliations:** a Centre for Medical Humanities and School of Medicine, Pharmacy and Health, Durham University, Durham, UK; b Department of Psychology, Durham University, Durham, UK; c Centre for Medical Humanities and Department of Geography, Durham University, Durham, UK; d Department of Anthropology, Stanford University, Stanford, CA, USA; e Lived Experience Research Network, Baltimore, MD, USA

## Abstract

**Background:**

Auditory hallucinations—or voices—are a common feature of many psychiatric disorders and are also experienced by individuals with no psychiatric history. Understanding of the variation in subjective experiences of hallucination is central to psychiatry, yet systematic empirical research on the phenomenology of auditory hallucinations remains scarce. We aimed to record a detailed and diverse collection of experiences, in the words of the people who hear voices themselves.

**Methods:**

We made a 13 item questionnaire available online for 3 months. To elicit phenomenologically rich data, we designed a combination of open-ended and closed-ended questions, which drew on service-user perspectives and approaches from phenomenological psychiatry, psychology, and medical humanities. We invited people aged 16–84 years with experience of voice-hearing to take part via an advertisement circulated through clinical networks, hearing voices groups, and other mental health forums. We combined qualitative and quantitative methods, and used inductive thematic analysis to code the data and χ^2^ tests to test additional associations of selected codes.

**Findings:**

Between Sept 9 and Nov 29, 2013, 153 participants completed the study. Most participants described hearing multiple voices (124 [81%] of 153 individuals) with characterful qualities (106 [69%] individuals). Less than half of the participants reported hearing literally auditory voices—70 (46%) individuals reported either thought-like or mixed experiences. 101 (66%) participants reported bodily sensations while they heard voices, and these sensations were significantly associated with experiences of abusive or violent voices (p=0·024). Although fear, anxiety, depression, and stress were often associated with voices, 48 (31%) participants reported positive emotions and 49 (32%) reported neutral emotions. Our statistical analysis showed that mixed voices were more likely to have changed over time (p=0·030), be internally located (p=0·010), and be conversational in nature (p=0·010).

**Interpretation:**

This study is, to our knowledge, the largest mixed-methods investigation of auditory hallucination phenomenology so far. Our survey was completed by a diverse sample of people who hear voices with various diagnoses and clinical histories. Our findings both overlap with past large-sample investigations of auditory hallucination and suggest potentially important new findings about the association between acoustic perception and thought, somatic and multisensorial features of auditory hallucinations, and the link between auditory hallucinations and characterological entities.

**Funding:**

Wellcome Trust.

## Introduction

Auditory hallucinations—or voices—are a common feature of schizophrenia. They also occur in other disorders and in individuals with no psychiatric history.^1^ Understanding of subjective experiences of hallucination—and how they vary between different populations—is a central concern of psychiatry, and can help with the development of new causal accounts of auditory hallucination and more effective therapeutic interventions.^2,3^

Although various resources document first-person experiences of voice-hearing,^4^ systematic empirical research on the phenomenology of auditory hallucinations remains scarce. Nayani and David's 1996 study^5^ analysed clinical interview data from 100 patients with psychosis with auditory hallucinations (61% of 100 individuals had ICD-10 schizophrenia diagnoses). The investigators concluded that auditory hallucinations in this population are typically repetitive emotive utterances that increase in number and complexity over time. In 2014, McCarthy-Jones and colleagues^6^ analysed auditory hallucination descriptions from 199 patients (81% of individuals had a diagnosis of DSM-III-R schizophrenia), obtained through the Mental Health Research Institute (MHRI) Unusual Perceptions Scale.^7^ Cluster analysis of these findings suggested four common factors: voices that were repetitive, commanding or involved running commentary (86%); voices similar to a person's own thoughts (36%); voices that were clearly reminiscent of specific memories (12%); and non-verbal auditory hallucinations (42%).^6^

Although such surveys provide insight into the experience of auditory hallucinations, the focus on psychosis, particularly schizophrenia, leaves the potential cross-diagnostic features of auditory hallucinations unexplored. Additionally, the semi-structured interviews and closed-ended approaches often used make several a priori assumptions about the key features of auditory hallucinations, which prioritise some structural characteristics (eg, loudness) over others (eg, voice identity). Clinical terminology is often itself loaded and might prime or encourage participants to describe their experiences in particular ways (eg, as auditory or linguistic). From a phenomenological perspective, these approaches might constrain understanding of auditory hallucinations in potentially serious ways.^8,9^

To address these concerns, and as part of the Hearing the Voice project and Lived Experience Network, we developed a questionnaire on voices and voice-like experiences. We drew on the expertise of philosophers, psychologists, medical humanities scholars, and researchers with lived experience of auditory hallucination, in consultation with clinicians and people who hear voices, from the project's advisory group. We aimed to record a detailed and diverse collection of experiences, in the words of the people who hear voices themselves.

## Methods

### Participants

We made the questionnaire available via the project website for 3 months for anonymous online completion. We invited people aged 16–84 years with experience of voice-hearing to take part via an advertisement circulated through clinical networks, hearing voices groups, and other mental health forums. We asked participants if they had ever received a psychiatric diagnosis, and if so, to report their present or most recent diagnosis. Participants consented to use of their data in the study before accessing the questionnaire and confirmed this upon completion. All procedures were approved by Durham University ethics committee.

### Procedures

Participants completed a 13 item questionnaire that was available online through Qualtrics (Provo, UT, USA; appendix). Recognising that no term is neutral or universally accepted, we chose to use the term voices because it is widely understood and used in non-clinical and clinical contexts. Many people who hear voices regard the term auditory hallucination as stigmatising because it implies that their experiences are not real.^10,11^ Furthermore, we did not want to restrict the study by implying that the phenomena in question are necessarily always auditory or perceptual. We designed the questions to be unbiased, non-leading, and non-hierarchising prompts that aimed to elicit phenomenologically rich data. The questionnaire combined closed-ended and open-ended questions (eg, “Please try to describe your voice(s) and/or voice-like experiences”; “How, if at all, are these experiences different from your own thoughts?”). All questions were optional and no word limit was imposed on responses.

### Statistical analysis

We analysed the data using a mixture of qualitative and quantitative methods. First, we integrated responses into single narratives. We then did an inductive thematic analysis.^12,13^ Each member of the research team initially coded 20 responses. Once collated, we refined and organised the lists of codes into a coding framework with inclusion and exclusion criteria noted for each code. Two independent raters (AW and NJ) then coded the data using NVivo 10 software. Once high inter-rater reliability (κ=0·85) was established for 30% of the sample, the raters divided and coded the remaining data independently. Responses were analysed as single integrated narratives that could be assigned each code a maximum of once. Any ambiguous instances were resolved through discussion and a consensus-based decision.

The nature of some questions allowed for mutually exclusive categorical coding of responses (eg, codes for child, adolescent, and adult onset). However, most of the codes that we used were not mutually exclusive because participants often described a range of phenomenological and structural characteristics.

We used coded data to calculate descriptive statistics for common features of voice-hearing across the full sample. We used a mixed-methods priority-sequence model, in which we used quantitative analyses (χ^2^ tests) to test additional associations of selected codes that were either identified in the principal qualitative analyses or suggested by previous studies.^14^ We applied a false discovery rate correction^15^ to correct for multiple comparisons. We did not calculate any post-hoc measures of power for the study, mainly because specific hypothesis testing was not the focus of the study (as this would contradict key components of the phenomenological method), but also because of theoretical concerns about the notion of post-hoc power.

### Role of the funding source

The funder of the study had no role in study design, data collection, data analysis, data interpretation, or writing of the report. The corresponding author had full access to all the data in the study and had final responsibility for the decision to submit for publication.

## Results

157 participants completed the survey, and we excluded four responses that did not discuss voice-hearing experiences, for a total of 153 responses. Various diagnoses were reported (table 1), the most common of which were schizoaffective disorder (24 [16%] of 153 individuals) and bipolar disorder (21 [14%] individuals). The total length of the responses ranged from 24 to 2474 words (mean 510 words, SD 432). Table 2 shows demographic details of the survey population.

Less than half of participants described literally auditory experiences (ie, voices indistinguishable from voices or other sounds), and 14 (9%) individuals reported exclusively thought-like voices (ie, with no auditory qualities; table 3). We encouraged description of the differences in the characteristics of these experiences (panel 1) by using questions that directly invited participants to compare voices with their thoughts and actual voices in the room (appendix). 56 (37%) participants—coded as auditory–thought mixed—reported either a combination of auditory and thought-like voices or experiences that were somewhere between literally auditory and thought-like.

Notably, most individuals who described their experiences as non-literally auditory still referred to them as voices. About a fifth (30 individuals) of the sample deemed voice an inadequate term for their experience, instead using terms such as “intuitive knowing” or “telepathic experience”, or descriptors such as “alters”, “parts”, or “fellow system members”.

124 (81%) participants reported the presence of several voices, with only 10 (7%) individuals reporting a single voice. Most participants reported having had multiple voices, with a quarter (39 individuals) reporting undifferentiated or ambiguous collections of voices, such as crowds, gangs, or classroom groups. Voices with a physical location were equally likely to be external or internal.

Most voices were described as being characterful in some way (table 4)—ie, people or person-like entities with distinct characteristics, such as gender, age, patterned emotional responses, or intentions.

“I hear distinct voices. Each voice has their own personality. They often try to tell me what to do or try to interject their own thoughts or feelings about a certain subject or matter […] My voices range in age and maturity. Many of them have identified themselves and given themselves names.”

“I hear a mixture of men and women, but no children. They usually tell me to do things, but not dangerous things. Like they'll tell me to take out the garbage or check the lock on the window or call someone. Sometimes they comment on what I'm doing and whether I'm doing a good job or what I could be doing better.”

Roughly a fifth (33 [22%] of 153) of participants described voices that were recognised as specific, existing individuals. 24 (16%) participants described voices that were understood to be supernatural or spiritual entities.

Common characteristics of address were conversational voices (engaging the voice-hearer directly) or voices that commented on specific things. Few people reported only so-called simple voices—single words or brief phrases—or voices that did not address them directly. Only 8 (5%) participants reported voices which predominantly issued negative commands; overall experiences of abusive or violent voices were much more common.

Although many voices were described as either positive or neutral in tone, negative emotions were often associated with them, especially fear, anxiety, depression, and stress.

“Starting when I was about 20 years old, I heard the voices of demons screaming at me, telling me that I was damned, that God hated me, and that I was going to hell… The voices were so frightening and disruptive that much of the time I was unable to focus or concentrate on anything else.”

“To a point, they generally are anything but kind to me. They can be brutally sarcastic and intrusive.”

About two-thirds of participants (101 individuals) reported changes in bodily experience when they heard voices (table 4), which varied substantially.

“My body and brain felt like they were on fire when I heard the voices; I had constant tingling sensations throughout my extremities and shock-like sensations in my solar plexus.”

“Yes, my body felt more distant from me—the whole experience felt a bit dreamlike (like living a dream), surreal, other worldly.”

“At the very beginning I experienced a heat and a strong irritation in the right frontal part of my brain.”

28 (18%) people had multisensory voices, suggesting that their voices were perceived simultaneously through more than one sensory modality. 43 (28%) participants reported distinct hallucinations in other senses, and some people also described voices that gave access to other minds, or information that would not otherwise be available. A few (10–20) participants reported experiences of tiredness, sleep disturbance, and mania.

In cases where participants described their first voice experiences, the experiences often occurred in childhood (table 5). Many participants reported negative or explicitly traumatic circumstances, with few voices (17 [11%] of 153 individuals) arising in positive or neutral circumstances. More than a third (53 of 153 individuals) of participants described structural transformations in the number and presence of voices over time, with a few (19 [12%] individuals) also reporting changes in voice content, frequency, or valence (emotional reaction elicited). Only one respondent specifically stated that their voice had not changed over time. Although 34 (22%) participants stated that they were unable to influence their voices, 54 (35%) reported that they could influence their voices indirectly (through strategies of avoidance, medication, or environmental change), and 69 (45%) individuals reported influencing their voices by engaging directly with them or exploring their meaning. The effect of the voices on participants' relationships with others was largely negative: 48 (31%) participants cited direct negative effects (eg, voices interrupting conversation or making it difficult to understand what others were saying), and 61 participants (40%) referenced a general negative effect, including experiences of stigma, fear, and loneliness.

To investigate the distinction between auditory and mixed auditory and thought-like voices, we compared numbers of people reporting each type of voice for a selection of the codes identified during the qualitative analysis (table 6). Participants with mixed auditory and thought-like voices were more likely than those with purely auditory experiences to report voices that were internal (p=0·010), conversational (p=0·010), had changed over time (p=0·030), and gave access to other minds (p=0·026). Mixed voices trended non-significantly towards being associated with voices that gave access to information that was otherwise unknown by the participant (p=0·051). No other contrasts were significant (table 6).

We compared participants with and without characterful voices (table 7). People who heard characterful voices were significantly more likely to be able to influence their voices (p=0·040) and, at the non-significant trend level, were more likely to experience voices that were abusive or violent (p=0·051) than were those who heard non-characterful voices (table 7).

We compared participants who specifically reported effects on the body with those who did not (table 8). Participants with bodily experiences were more likely to report voices that were abusive or violent (p=0·024) and to be able to anticipate their voices (p=0·025) than were those with no bodily effect. Reporting of bodily experiences seemed to be associated with reporting of traumatic circumstances when participants first heard voices, voices that were associated with shame, and few positive and useful voices (p=0·05–0·06; table 8).

A unique characteristic of our sample was its cross-diagnostic nature, including some participants who specifically reported that they had never received a psychiatric diagnosis (26 [17%] of 153 individuals). Based on previous research with similar populations,^16–18^ we compared people who had received a clinical diagnosis with those who had not (table 9). Participants who had not been clinically diagnosed were significantly less likely to associate their voices with fear (p=0·010) or depression (p=0·015) than were those with a clinical diagnosis. We detected no differences for any other categories (table 9).

To help with comparison with previous studies, we also did an exploratory analysis to compare participants who reported schizophrenia-related diagnoses (schizophrenia or schizoaffective disorder, n=38) with all other participants for a selection of codes associated with the classic understanding of auditory hallucinations in schizophrenia as auditory, externally located, and commanding phenomena. We identified no significant differences, even if we used an uncorrected p value cutoff (codes used: auditory, auditory-thought mixed, internal location, external location, single voice, multiple voices, and commanding nature).

The sample of respondents included a large proportion of female participants. To check for the effect of gender, we did χ^2^ analyses to compare men and women for group membership in the four subgroups analysed (auditory voices, characterful voices, bodily effect, and clinical diagnosis), and in association with all codes analysed (to avoid type II errors, we did not apply a false discovery rate correction). We detected no significant associations between gender and subgroup, and only three codes were significantly associated: paranoia was more likely in men (p=0·036), shame (p=0·022), and stress (p=0·006) women. However, the relative percentage of women did noticeably vary between diagnostic groups (appendix).

## Discussion

We used an open-ended, internet-based survey to obtain detailed information about the phenomenology of auditory hallucination from a diverse array of individuals, including those without psychiatric diagnoses (panel 2). Several of our findings are consistent with other large-sample studies of auditory hallucinations^5,6,18,19^ and longstanding clinical observations—ie, the high prevalence of multiple voices, typically with distinct characteristics; variations in acoustic properties, linguistic complexity and location; and strong associations with negative emotion, especially for individuals with psychiatric diagnoses.^5,6,20–22^

However, unlike the published scientific literature, our findings also suggest novel and under-researched aspects of auditory hallucination phenomenology. Specifically, we focus on distinctions between thought-like, mixed, and strictly auditory voices; voices with somatic effects; and the experiential complexities of characterful voices.

Although auditory hallucinations are usually understood as predominantly perceptual experiences, nearly half of our participants described their voices either as thought-like or as having both auditory and thought-like qualities. Such mixed voices were significantly more likely to be conversational, show change over time, and be experienced as giving access to other minds. So-called sound talk (mentions of loudness, timbre, pitch, resonance, accent, and rhythm) was very common throughout the sample, complicating clear distinctions between thoughts and perceptions (eg, “My thoughts are shouting” or “I experience a silent scream […] a presence, an emotional energy, or potential that I can feel but not hear”). These findings are similar to historical, cognitive, and phenomenological research on the qualities of imagined sound^23,24^ and raise the question of whether some voices might be better understood as passive or uncontrolled imagined perceptions, rather than perceptual hallucinations. The extent to which the message of auditory hallucinations can be understood without being heard is also worthy of further study.

Participants also frequently reported multisensory voices, concurrent somatic events, and hallucinations in other sensory modalities. Whether we classify these other-sensory or somatic features as adjunctive components of auditory hallucinations or instead as events distinct from specifically auditory hallucinations, the implications of our findings are potentially important to attempts to understand and assign subtypes to hallucinatory phenomena. The high prevalence of multisensory voices and somatic features is also important in view of the scarce attention to such features in existing clinical interventions, and could inform further development of theoretical models that link self-recognition to deficits in sensory-motor control at the level of body schema.^25^ Notably, voices with effects on the body were also significantly more likely be associated with an overall experience of voices that were abusive or violent, and voices that could be anticipated in some way. Although we did not detect a significant association between voices with somatic aspects and trauma, the strong associations between abusive voices and childhood adversity,^26^ especially sexual and physical trauma,^27^ suggest that this association might be promising for future study.

Command hallucinations are widely regarded as distressing and indicative of high risk of harm to self and others,^28^ and yet their content, severity, and importance have tended to be assumed rather than fully investigated. Command hallucinations were reported by 84% of 100 participants in Nayani and David's study^5^ and “constant, commanding and commenting” auditory hallucinations were reported by 86% of 199 participants in McCarthy-Jones and colleagues' study.^6^ We coded voices that issued negative commands or instructions to do harmful things as commanding, distinct from voices that issued requests or instructions to do things that were benign or helpful. Thus defined, command hallucinations characterised the overall experience of voice-hearing for only 8 (5%) of 153 participants. This discrepancy between our study and other phenomenological surveys could be caused by differences in populations and settings between studies: command hallucinations might be the dominant experience for individuals with a schizophrenia diagnosis, or those who are reporting on their voices in a clinical context and engaging with health-care services. Alternatively, a substantial number of people who hear voices who receive advice or strong suggestions from their voices might have been mislabelled as experiencing commands that are presumed to be inherently violent or potentially harmful.

The characterful or person-like nature of voices has been widely documented,^4,10^ is directly addressed by existing psychological interventions for voices,^29^ and was one of the most common aspects of voice-hearing reported in our analysis. However, little investigation has been done on the different ways that voices might be experienced as personified. The descriptions in our data suggest a range of person-like qualities, from amorphous entitativity (an undefined disembodied personality), to stereotypical person-like presentations (an angry man, an old woman), spiritual entities with anthropomorphic traits, specifically recognisable individuals, and voices that are subjectively experienced as representing all or part of the person's own self. Characterful voices were also distinguishable from other voices in their susceptibility to influence by the voice-hearer: more characterful voices could be directly engaged with in a meaningful way. These findings raise important conceptual, philosophical, and clinical questions for future research, including how the characterological features of voices are shaped by individuals' explanatory beliefs and local cultures.^30^ The heterogeneity of characterful voices also underscores the importance of existing relational interventions^29,31^ to address variability in the types of voices and their person-like qualities.

One limitation of the present study was the coding of characteristics derived from free-text written responses; some participants might have had particular experiences (such as command hallucinations), but not independently volunteered this information in our questionnaire. Our results might therefore underestimate the prevalence of features we coded for. Conversely, characteristics that are routinely discussed in clinical settings (such as voice location) might have been over-represented compared with less studied aspects of auditory hallucination experience. Ultimately, phenomenological investigation provides “no means to check the ‘truth’ of the responses recorded”, as noted by Nayani and David,^5^ and the departure from psychometrically validated measures limits the extent to which comparisons can be drawn between this study and other studies of auditory hallucination phenomenology. However, adoption of an exploratory, rather than prescriptive, approach to what counts as a voice or voice-like experience yields new insights into what people who hear voices themselves regard as most important. These insights are potentially of great importance to existing research frameworks that depend on assumptions that our data call into question, such as a focus on auditory hallucination as a primarily perceptual event.

Second, the online questionnaire was accessible only to English-speakers with basic internet literacy and access. Although the online platform might be thought to limit participation, results from research have shown that people with severe mental illness have rates of smartphone access and usage similar to the general public.^32^ We mainly recruited to the study through existing research, clinical, and service-user networks. High-functioning users of social media who are already engaged in such networks or communities might be over-represented, while individuals who are currently in acute care settings are almost certainly under-represented. Moreover, although the capacity to participate anonymously might have encouraged frank responses from some participants, we were unable to verify participants' self-reports. Because these self-reports include self-reported diagnoses, we have restricted ourselves to clinical versus non-clinical diagnoses and schizophrenia-spectrum versus other comparisons, rather than more specific distinctions between clinical diagnoses. In-depth comparison of voice phenomenology in different diagnostic contexts—including dissociative identity disorder and post-traumatic stress disorder—is a crucial topic for future studies of this kind.

Third, our overall sample shows substantial bias in terms of gender and ethnicity, limiting the representativeness and generalisability of our findings. 2·5 times as many women as men completed the study, which might be indicative of wider trends in survey response rates^33^ and hallucination proneness,^34^ and the cross-diagnostic nature of our sample. Although people from black and minority ethnic origins are up to nine times more likely than people from other ethnic origins to present with symptoms of psychosis,^35^ they were under-represented in our study. When we analysed gender effects in our data, we detected differences for only three codes: paranoia (which was more likely in men), childhood onset, and structured longitudinal change (which were both more likely in women than in men). These results might be caused by differences worthy of future attention, but their exploratory nature makes these findings tentative at best.

Despite these limitations, our methods allowed us to reach a demographically and diagnostically diverse sample, which included participants with little or no current contact with mental health services. The use of more prescriptive clinical tools, or confining of our sample to clinical settings, would possibly have limited the range of experiences reported. If full understanding of the phenomenology of auditory hallucination is important, across diagnoses and between clinical and non-clinical populations,^2,21^ then such methods are a necessary starting point.

By engaging a sample of people who hear voices with varying diagnoses and clinical histories, we report both overlap with past qualitative investigations of auditory hallucination and potentially important new findings that depart from previous studies of the phenomenology of voices. These findings underscore the importance of future investigations of the association between acoustic perception and thought, the somatic and multisensorial features of auditory hallucination, and the link between auditory hallucination and characterological entities.

**This online publication has been corrected. The corrected version first appeared at thelancet.com/psychiatry on April 2, 2015**

## Figures and Tables

**Table 1:** Diagnostic information by gender

	**Female (n=100)**	**Male (n=40)**	Other*(n=13)
Schizoaffective disorder	14 (9%)	9 (6%)	1 (1%)
Bipolar disorder	16 (10%)	5 (3%)	0
Major depression	11 (7%)	2 (1%)	1 (1%)
Schizophrenia	5 (3%)	9 (6%)	0
Post-traumatic stress disorder	9 (6%)	1 (1%)	1 (1%)
Dissociative identity disorder	7 (5%)	0	4 (3%)
Borderline personality disorder	5 (3%)	2 (1%)	1 (1%)
Depression (mixed)	4 (3%)	2 (1%)	1 (1%)
Generalised anxiety disorder	5 (3%)	0	1 (1%)
Psychosis (NOS)	2 (1%)	1 (1%)	1 (1%)
Obsessive compulsive disorder	1 (1%)	1 (1%)	1 (1%)
Other diagnosis	3 (2%)	1 (1%)	1 (1%)
No diagnosis	18 (12%)	7 (5%)	1 (1%)

Not all patients gave all details, therefore percentages do not always sum to 100%. NOS=not otherwise specified.

* Other includes androgyny, genderfluid, genderqueer, transgender, non-binary, and bigender.

**Table 2:** Demographic information

	**Number of participants (n=153)**
**Country**	
UK	48 (31%)
USA	76 (50%)
Australia	9 (6%)
Canada	7 (5%)
Other	13 (8%)
Ethnic origin*	
White	106 (69%)
Mixed-race	16 (10%)
Country-defined	13 (8%)
Black or ethnic minority	9 (6%)
Other	3 (2%)
Not specified	6 (4%)
Sexuality*	
Heterosexual	89 (58%)
Bisexual	19 (12%)
Homosexual, gay, or lesbian	13 (8%)
Queer or pansexual	10 (7%)
Asexual	9 (6%)
Other	2 (1%)
Not specified	11 (7%)
Religious beliefs*	
Christian	45 (29%)
None or atheist	44 (29%)
Spiritual or mixed	9 (6%)
Pagan or pantheistic	8 (5%)
Buddhist	4 (3%)
Jewish	2 (1%)
Other	7 (5%)
Not specified	34 (22%)
**How did you hear about the study?**	
Social media (Twitter, Tumblr, Facebook)	32 (21%)
Hearing the Voice project	27 (18%)
Referred by a friend	24 (16%)
Other (unspecified)	21 (14%)
Mental health forum or blog	18 (12%)
Referred by a mental health professional	11 (7%)
Lived Experience Research Network	10 (7%)
Intervoice	7 (5%)
Newspaper article	6 (4%)
Other hearing voices groups	3 (2%)

Not all patients gave all details, therefore percentages do not always sum to 100%.

* Codes derived from free-text responses.

**Table 3:** Nature and location of voices

	**Number of participants (n=153)**
Auditory*	67 (44%)
Thought-like*	14 (9%)
Mixed auditory or thought-like*	56 (37%)
External	69 (45%)
Internal	67 (44%)
Single*	10 (7%)
Multiple*	124 (81%)
Undifferentiated voices	39 (25%)
Voice as inadequate description	30 (20%)

Data are n (%). Not all patients gave all details, therefore percentages do not always sum to 100%.

* Mutually exclusive categorical codes.

**Table 4:** Character, emotion, experiences associated with voices

	**Number of participants (n=153)**
**Characteristics**	
Characterful*	106 (69%)
Not characterful*	22 (14%)
Recognised individual	33 (22%)
Supernatural entity	24 (16%)
Simple address	16 (10%)
No direct address	16 (10%)
Commenting voices	18 (12%)
Conversational voices	56 (37%)
Commanding voices	8 (5%)
Abusive and violent voices	54 (35%)
Positive and helpful voices	46 (30%)
Spiritual purpose	24 (16%)
**Emotions**	
Fear	63 (41%)
Positive	48 (31%)
Neutral	49 (32%)
Anxiety	47 (31%)
Depression	44 (29%)
Anger	32 (21%)
Stress	26 (17%)
Suicidal	26 (17%)
Sadness	21 (14%)
Shame	21 (14%)
Loneliness	16 (10%)
**Other kinds of experiences**	
Bodily effect*	101 (66%)
No bodily effect*	41 (27%)
Tiredness	10 (7%)
Sleep disturbance	20 (13%)
Mania	13 (8%)
Paranoia	23 (15%)
Musical	17 (11%)
Non-verbal	21 (14%)
Other hallucinations	43 (28%)
Multisensory	28 (18%)
Access to other minds	21 (14%)
Access to other information	19 (12%)

Data are n (%). Not all patients gave all details, therefore percentages do not always sum to 100%.

* Mutually exclusive categorical codes.

**Table 5:** Causes and effects of voices

	**Number of participants (n=153)**
**Voice onset**	
Child*	52 (34%)
Adolescent*	32 (21%)
Adult*	29 (19%)
**Circumstances**	
Positive	17 (11%)
Negative	36 (24%)
Traumatic	35 (23%)
Substance use	10 (7%)
**Change, influence, and anticipation**	
Structured change to voices	53 (35%)
Change within a voice	19 (12%)
**Influence**	
Can influence directly	69 (45%)
Can influence indirectly	54 (35%)
Cannot influence	34 (22%)
**Anticipation**	
Can generally anticipate	32 (21%)
Can specifically anticipate	35 (23%)
Cannot anticipate	70 (46%)
Continuous voices	22 (14%)
**Effect on personal relationships**	
General negative effect	61 (40%)
Direct negative effect	48 (31%)
Positive effect	14 (9%)
No effect	42 (27%)

Data are n (%). Not all patients gave all details, therefore percentages do not always sum to 100%.

* Mutually exclusive categorical codes.

**Table 6:** Characteristics of voice-hearing associated with type of nature of voices

	**Auditory voices (n=67)**	**Mixed voices (n=56)**
Internal location*	19 (28%)	33 (59%)
External location	34 (51%)	28 (50%)
Multisensory	8 (12%)	12 (21%)
Conversational*	18 (27%)	31 (55%)
Direct influence	25 (37%)	30 (54%)
Structured longitudinal change*	19 (28%)	29 (52%)
Access to other minds*	4 (6%)	13 (23%)
Access to information	4 (6%)	11 (20%)
Bodily effect	40 (60%)	41 (73%)

Data are n (%). Percentages are for participants within a subgroup receiving that code. Not all patients gave all details, therefore percentages do not always sum to 100%.

* Significant associations (all p<0·05, corrected for false discovery rate).

**Table 7:** Characteristics of voice-hearing associated with characterful voices

	**Characterful (n=106)**	**Not characterful (n=22)**
Direct influence*	60 (57%)	6 (27%)
Bodily effect	74 (70%)	15 (68%)
Abusive or violent	41 (39%)	3 (14%)
Fear	48 (45%)	5 (23%)
Anxiety	35 (33%)	6 (27%)
Depression	32 (30%)	5 (23%)

Data are n (%). Percentages are for participants within a subgroup receiving that code. Not all patients gave all details, therefore percentages do not always sum to 100%.

* Significant associations (all p<0·05, corrected for false discovery rate).

**Table 8:** Characteristics of voice-hearing associated with bodily effect

	**Bodily effect (n=101)**	**No bodily effect (n=41)**
Multisensory	21 (21%)	5 (12%)
Positive or useful	25 (25%)	18 (44%)
Abusive or violent*	43 (43%)	7 (17%)
Traumatic circumstances	28 (28%)	4 (10%)
Fear	47 (47%)	13 (32%)
Anxiety	35 (35%)	8 (20%)
Shame	17 (17%)	1 (2%)
Anticipation*	48 (48%)	9 (22%)

Data are n (%). Percentages are for participants within a subgroup receiving that code. Not all patients gave all details, therefore percentages do not always sum to 100%.

* Significant associations (all p<0·05, corrected for false discovery rate).

**Table 9:** Characteristics of voice-hearing associated with diagnosis

	**Clinical (n=127)**	**Non-clinical (n=26)**
Auditory	52 (41%)	15 (58%)
Positive and useful voices	34 (27%)	12 (46%)
Abusive and violent voices	49 (39%)	5 (19%)
Fear*	60 (47%)	3 (12%)
Anxiety	41 (32%)	6 (23%)
Depression*	43 (34%)	1 (4%)
Bodily effect	87 (69%)	14 (54%)

Data are n (%). Percentages are for participants within a subgroup receiving that code. Not all patients gave all details, therefore percentages do not always sum to 100%.

* Significant associations (all p<0·05, corrected for false discovery rate).

## Panel 1 Nature of experiences

**Auditory**

“[M]ost of the time I can hear it like it was just someone standing next to me. It's a different feeling than when you think words inside of your head, when you think inside your head your voice isn't distinct like it is when you speak out loud. You think words, not tone. But there is definite distinct tone and individuality that's unfamiliar with the voices.”

**Thought-like**

“I did not hear the voices aurally. They were much more intimate than that, and inescapable. It's hard to describe how I could ‘hear’ a voice that wasn't auditory; but the words the voices used and the emotions they contained (hatred and disgust) were completely clear, distinct, and unmistakable, maybe even more so than if I had heard them aurally.”

**Mixed**

“I have all kinds of voice-type experiences […] Some are voices that are clearly in my head but which feel ‘different’ from my own thoughts. Some are voices that seem to come from outside but which I know don't.”

## Panel 2 Research in context

**Systematic review**

Before constructing the survey, we did a systematic review of the published literature on hallucinations across diagnostic (and non-clinical) populations. We initially employed the search terms “phenomenology” and “hallucinations”— where possible also limiting the methods employed to “qualitative”—across the PsycINFO and PubMed databases. These searches returned 237 and 125 initial articles, respectively. Each article abstract was then reviewed individually; of those directly relevant to our project (ie, moderate to large-sample [n>50] phenomenological studies of auditory or verbal hallucinations), we searched cited references to identify any additional relevant articles, in addition to future articles that used the base article as a reference. We searched cited references until no additional articles of relevance could be identified. Although we were able to identify a sub-set of articles employing structured or semi-structured measures and comparing the phenomenology of hallucinations across specific diagnostic groups (eg, Parkinson's disease *vs* schizophrenia), we did not identify any published studies that simultaneously surveyed both clinical and non-clinical individuals; included individuals with any diagnosis (psychiatric, neurological, or medical); and used open-ended (unstructured) prompts.

**Interpretation**

We report the findings of what is, to our knowledge, the largest open-ended survey of the phenomenology of voices and voice-like events in the published scientific literature. We departed from other large-sample qualitative studies of auditory hallucination by targeting a diverse, naturalistic sample of individuals with and without clinical histories and with a broad range of (self-reported) diagnoses. Potentially important new findings concern the association between acoustic perception and thought, somatic and multisensorial features of auditory hallucinations, and the link between auditory hallucinations and characterological entities. Awareness and further investigation of these characteristics has substantial implications for experimental and applied clinical research programmes, especially with respect to further development of interventions targeting the way voice-hearers relate to their voices.

## Supplementary Material

Supplementary appendix

Supplementary audio What does it mean to hear voices? Authors Angela Woods and Ben Alderson-Day discuss the results of their new study with Lancet Psychiatry Editor Niall Boyce.
