# Corrections

**DOI:** 10.1016/S2215-0366(15)00163-7

**Published:** 2015-05

*Woods A, Jones N, Alderson-Day B, Callard F, Fernyhough C. Experiences of hearing voices: analysis of a novel phenomenological survey.* Lancet Psychiatry *2015;* ***2***: *323–31*—In table 1 of this Article (published Online First on March 10, 2015), values for the number of individuals of different genders have been corrected for schizoaffective disorder, schizophrenia, post-traumatic stress disorder, borderline personality disorder, and mixed depression. In the last paragraph of the Results, the third sentence should have read “We detected no significant associations between gender and subgroup, and only three codes were significantly associated: paranoia was more likely in men (p=0·036), while childhood onset (p=0·001) and structured longitudinal change (p=0·039) was more likely in women.” Additionally, the fourth sentence of the ninth paragraph in the Discussion should have read “When we analysed gender effects in our data, we detected differences for only three codes: paranoia (which was more likely in men), childhood onset, and structured longitudinal change (which were both more likely in women than in men).” The online version has been corrected as of April 2, 2015.

