# Supplementary material for: Experiences of hearing voices: analysis of a novel phenomenological survey
Source: Lancet Psychiatry. 2015 Apr;2(4):323–31. doi: 10.1016/S2215-0366(15)00006-1 (PMC4580735; doi:10.1016/S2215-0366(15)00006-1)
Supplement: Supplementary appendix [file mmc1.pdf]

THE LANCET Psychiatry

Supplementary appendix

This appendix formed part of the original submission and has been peer reviewed. We post it as supplied by the authors.

Supplement to: Woods A, Jones N, Alderson-Day B, Callard F, Fernyhough C. Experiences of hearing voices: analysis of a novel phenomenological survey. *Lancet Psychiatry* 2015; published online March 11. http://dx.doi.org/10.1016/S2215-0366(15)00006-1.

Supplementary Materials

Questionnaire Items

Some people find the term “hearing voices” a useful way to describe their experience. Others hear sounds but not speech, describe forms of telepathy and loud thoughts, or feel the presence of other beings. Our aim is to develop a better understanding of all of these experiences in terms which are meaningful to you, so please feel free to use whatever language you’re most comfortable with.

1. Please try to describe your voices(s) and/or voice-like experiences.
   How, if at all, are these experiences different from your own thoughts?
2. How, if at all, are these experiences different from hearing the voice of someone who is present in the room?
3. Does it feel as though the voice(s) that you hear have their own character or personality?
4. Can you tell us a bit about what life was like for you, and how you were feeling, when you first started hearing voices or having voice-like experiences?
5. What kinds of moods or emotions are associated with your voices?
6. Does your body feel different when you experience voices?
7. Do you know when you are about to experience a voice? If so, how?
8. How, if at all, do your voices affect your relations with other people?
9. If you have taken medication, how has this affected your voices?
10. Over time, have you been able to influence your voices? If so, how?
11. What other kinds of experience, if any, accompany your experiences of voices?
12. Are there any aspects of your experience of hearing voices that this questionnaire has not covered? If so, please tell us about them here.

Supplementary table: **Participants’ genders by age groups**

	16–17 years	18–24 years	25–34 years	35–44 years	45–54 years	55–64 years	65–74 years
**Female**	6 (4%)	17 (11%)	18 (12%)	18 (12%)	20 (13%)	15 (10%)	6 (4%)
**Male**	1 (1%)	8 (5%)	8 (5%)	7 (5%)	8 (5%)	7 (5%)	1 (1%)
**Other***	4 (3%)	4 (3%)	5 (3%)	0 (0%)	0 (0%)	0 (0%)	0 (0%)

Data are number of participants (%). Not all patients gave all details, therefore percentages do not always add up to 100%. *Code derived from free-text responses, which included androgyny, genderfluid, genderqueer, transgender, non-binary, and bigender.
